# Serum PCSK9 levels, but not PCSK9 polymorphisms, are associated with CAD risk and lipid profiles in southern Chinese Han population

**DOI:** 10.1186/s12944-018-0859-5

**Published:** 2018-09-11

**Authors:** Gaojun Cai, Lei Yu, Zhiying Huang, Li Li, Xingli Fu

**Affiliations:** 1 Department of Cardiology, WujinHospital affiliated with Jiangsu University, Changzhou, Jiangsu Province, 213017 China; 2 Department of Pediatrics, No. 2 Hospital of Changzhou, Changzhou, 213001 Jiangsu China; 3 Jiangsu University Health Science Center, Zhenjiang, Jiangsu Province, 212001 China

**Keywords:** Lipids, Dyslipidaemia, Coronary artery disease, Gene, Polymorphism, Atherogenic index of plasma

## Abstract

**Background:**

Genetic and environment factors affect the occurrence and development of coronary artery disease (CAD). Proprotein convertase subtilisin/kexin type 9 (PCSK9), has been investigated extensively in the field of lipid metabolism and CAD. We performed this case-control study to investigate the relationship between serum PCSK9 levels and *PCSK9* polymorphisms and lipid levels and CAD risk in a southern Chinese population.

**Methods:**

A hospital-based case-control study with 1, 096 subjects, including 626 CAD patients and 470 controls, were conducted. Genotyping of *PCSK9* polymorphisms was performed using polymerase chain reaction-ligase detection reaction (PCR-LDR) method.

**Results:**

The frequencies of the AA, AG and GG genotypes of *PCSK9* E670G polymorphism were 90.58, 9.27, and 0.16% in the CAD patients, compared with 88.72, 10.85 and 0.43% in the controls, respectively. No R46L variant was detected in this population. There were no significant differences in genotype and allele frequencies of *PCSK9*E670G polymorphism between the CAD group and the controls. Serum lipid levels were not significantly different in carriers with the G allele and those with the AA genotype. The median (QR) of PCSK9 concentration was 1205.00 ng/l (577.28–1694.13 ng/l) in cases and 565.87 ng/l (357.17–967.50 ng/l) in controls, respectively. Compared with controls, CAD patients had significantly higher PCSK9 levels (z = 4.559, *P* < 0.001). After adjusting for age, gender, essential hypertension, diabetic mellitus, smoking and lipid profiles, PCSK9 levels remain significantly associated with increased CAD susceptibility (OR = 1.002, 95% CI = 1.001–1.002, *P* < 0.001). The correlation analyses showed that serum PCSK9 levels were positively associated with triglyceride (TG), Apo B and atherogenic index of plasma (AIP) levels in controls. No significant association between the *PCSK9* E670G polymorphism and serum PCSK9 levels was observed in the CAD group and the controls.

**Conclusions:**

The present study shows that serum PCSK9 levels, but not *PCSK9* polymorphisms, are associated with CAD risk in Southern Chinese Han population, and that serum PCSK9 levels are positively associated with AIP.

**Electronic supplementary material:**

The online version of this article (10.1186/s12944-018-0859-5) contains supplementary material, which is available to authorized users.

## Background

Coronary artery disease (CAD), which is characterized as multi-factor disease remains the leading cause of morbidity and mortality worldwide [1]. Genetic and environmental factors affect the occurrence and development of CAD. Numerous epidemiological and clinical studies have shown that dyslipidemia, including the high low-density lipoprotein cholesterol (LDL-C) and low high-density lipoprotein cholesterol (HDL-C) levels, is strongly associated with an increased risk of CAD [2]. Atherogenic index of plasma (AIP), a new comprehensive lipid index, comprehensively reflects the balance between atherogenic and anti-atherogenic factors, and is a stronger and independent risk factor for CAD [3, 4].

Proprotein convertase subtilisin/kexin type 9 (PCSK9), the ninth member of the proprotein convertase family, has been investigated extensively in the field of lipid metabolism [5]. By combining with the epidermal growth factor-like repeat A domain (EGF-A) of low-density lipoprotein receptor (LDLR) and lowing the recycling of LDLR to cell surface, PCSK9 mediates serum LDL-C levels. Recently, several PCSK9 inhibitors, such as alirocumab and evolocumab, were applied in patients with familial hypercholesterolemia and/or CAD in different countries, and were verified to decrease total cholesterol (TC), LDL-C, triglyceride (TG) and the ratio of TC to high-density lipoprotein cholesterol (HDL-C) greatly [6].

*PCSK9* gene is located on chromosome 1p32.3 and encompasses 12 exons. Until now, approximately 160 mutations in *PCSK9* gene have been investigated. According to their effects on LDLR, *PCSK9* gene mutations are divided into loss-of-function (LOF) and gain-of-function (GOF) mutations. LOF mutations in the *PCSK9* gene decrease LDLR degradation, thereby reducing serum LDL-C levels and the risk of CAD. Conversely, GOF mutations may increase serum LDL-C levels and CAD risk. *PCSK9* E670G polymorphism (rs505151) is a common variant and is a GOF mutation, while R46L (rs11591147) is a rare variant and belongs to the LOF mutations. Several studies have investigated the relationships between these two variants and lipid levels and CAD susceptibility in different populations. However, the results were inconsistent. In 2015 and 2017, three meta-analyses were carried out to clarify the association between *PCSK9* E670G polymorphism and lipid levels and the risk of CAD [7–9]. Although pooled effects indicated that G allele carriers had a higher risk of CAD and LDL-C levels than non-carriers, the heterogeneity among the studies was statistically significant, suggested that the results should be interpreted cautiously. Additionally, the relationships between the E670G polymorphism and other lipid profiles remained inconsistent. A study with a large sample size is needed to verify the results. Data from previous studies indicated that the variant of R46L is rare in both Caucasians and Asians [10, 11]. Caucasians carriers of the minor allele had lower cardiovascular disease susceptibility and lower LDL-C levels [9]. However, no study has been conducted on the relationship between the *PCSK9* R46L polymorphism and lipid levels and cardiovascular risk in a Chinese population. Moreover, no study has been designed to explore the relationship between PCSK9 and the comprehensive lipid index. Therefore, we carried out a hospital-based case-control study with 1, 096 subjects to investigate the relationship between *PCSK9* polymorphisms and lipid levels and CAD risk in a Southern Chinese population.

## Methods

### Subjects

The present retrospective case-control study included 1, 096 participants. All participants were Han Chinese and underwent coronary angiography (CAG) examination in the Department of Cardiology inWujin hospital affiliated to Jiangsu University between September 2012 and July 2017. Total of 626 CAD patients, including 364 myocardial infarction, 226 unstable angina pectoris and 36 stable angina pectoris involved in our study. The diagnosis of CAD was described in our previous study [12], which was in line with the World Health Organization criteria from 1978. The controls also underwent CAG examination in the same period and had a luminal stenosis of *<* 50% of the major coronary arteries. Judikin’s method was used in the CAG examination and the images were evaluated by two experienced cardiologists. Patients with advanced liver or kidney failure, malignant tumors, asthma and major surgery or trauma within one month were excluded. Patients currently using lipid-lowing drugs were also excluded in the present study. Informed consent was obtained from all participants. The study was approved by the ethics committee of Wujin hospital.

### Biochemical parameter analysis

Approximately 2 ml of venous blood was extracted from each participant after 12 h fasting. The methods to measure biochemical parameters were performed as previously described [13]. All of the biochemical parameters, including TC, TG, HDL-C, LDL-C, ApoA1, ApoB, Lp(a) and glucose levels, were analyzed by automatic biochemical analyzer (Olympus AU5400). AIP is calculated as log_10_ (TG/HDL-C).

### Genotyping of *PCSK9* polymorphisms

Total genomic DNA was extracted from peripheral blood leukocytes. The method of extraction was described in previous study [13]. Genotyping of *PCSK9* polymorphisms was performed using polymerase chain reaction-ligase detection reaction (PCR-LDR) method. The sequences of primers and probes are listed in Additional file 1: Table S1. PCR was performed with a volume of 25 μl reaction system, including 1 μl genomic DNA, 2 μl buffer, 0.6 μl Mg^2+^, 2 μl dNTP, 0.2 μl DNA polymerase, and 2 μl Primer mix. Multiplex PCR reaction was carried out by an initial denaturation at 95 °C for 2 min, followed by 40 cycles at 94 °C for 90 s, 53 °C for 1.5 min, 65 °C for 30 s, and a terminal extension 65 °C for 10 min. After PCR amplification, the LDR was carried out with a volume of 10 μl reaction system, including 4 μl PCR product, 1 μl buffer, 1 μl Probe mix, 0.05 μl Taq DNA ligase, and 4 μl ddH_2_O with a produce of 40 cycles of pre-denaturation at 95 °C for 2 min, annealing at 94 °C for 15 s, and extension at 50 °C for 25 s. The genotypes were analyzed by ABI PRISM 3730 sequencer and Genemapper software. The results of genemapper analyses are shown in Additional file 2: Figure S1.

### Statistical analysis

All data were analysed using SPSS 17.0 software package (SPSS Inc., Chicago, Illinois). The Hardy-Weinberg equilibrium (HWE) was assessed using the goodness-of-fit method. The Kolmogorov-Smirnov test was used to examine the normality of the distributions of continuous variables. For continuous variables that satisfied normal distribution, the data were expressed as the means ± standard deviation (SD) and were compared using independent-sample t-tests. Otherwise, the data were expressed as medians and quartile ranges (QR), and were compared using the Mann-Whitney test. Qualitative variables were assessed using the Chi- square test.The association between the genotypes and CAD risk was evaluated by calculating the values of the crude odds ratios (ORs), together with 95% confidence intervals (CIs) and adjusted ORs. Correlation analysis was performed using the Pearson test. A two-sided *p*-value of < 0.05 was regarded as significant.

## Results

### Characteristics of the involved participants

In total, 1, 096 subjects, including 626 CAD patients (439 males and 187 females; mean age, 64.18 ± 9.83 years) and 470 controls (243 males and 227 females; mean age, 61.62 ± 9.42 years), were enrolled in the study. As shown in Table 1, CAD patients were older than controls (*P* < 0.01). The frequencies of male gender, essential hypertension (EH), diabetes mellitus (DM) and smoking were significantly higher in the CAD patients than in the controls. In addition, the patients with CAD had higher TC, LDL-C and Apo B levels when compared with the control subjects. On the other hand, the levels of HDL-C and Apo A1 were significantly lower in the CAD patients than in the controls. In addition, there were no significant difference in TG levels between the CAD patients and the controls.

**Table 1:** The characteristics of CAD patients and controls

Characteristics	CAD (*n* = 626)	Controls (*n* = 470)	*χ*^2^(*t*)	*P*
Age (years)	64.18 ± 9.83	61.62 ± 9.42	4.337	< 0.01
Male [*n* (%)]	439 (70.13)	243 (51.70)	38.774	< 0.01
EH [*n* (%)]	454 (72.52)	276 (58.72)	22.986	< 0.01
DM [*n* (%)]	163 (26.04)	67 (14.26)	22.478	< 0.01
Smoking [*n* (%)]	239 (38.18)	114 (24.26)	23.835	< 0.01
TC (mmol/l)	4.59 ± 1.05	4.42 ± 0.96	2.807	< 0.01
TG (mmol/l)	1.84 ± 1.50	1.76 ± 1.46	0.949	> 0.05
HDL-C (mmol/l)	1.08 ± 0.27	1.16 ± 0.31	4.752	< 0.01
LDL-C (mmol/l)	2.93 ± 0.92	2.64 ± 0.79	5.572	< 0.01
ApoA1 (g/l)	1.18 ± 0.23	1.23 ± 0.24	3.691	< 0.01
ApoB (g/l)	1.16 ± 0.31	1.08 ± 0.27	3.789	< 0.01

*CAD* coronary artery disease, *TC* total cholesterol, *TG* triglyceride, *HDL-C* high-density lipoprotein cholesterol, *LDL-C* low-density lipoprotein cholesterol, *EH* essential hypertension, *DM* diabetes mellitus

### Association between the *PCSK9* polymorphisms and CAD risk

Table 2 shows that the genotype and allele frequencies of *PCSK9* E670G in the cases and controls. The genotype frequency in the controls was in accordance with HWE (*P* > 0.05). The frequencies of the AA, AG and GG genotypes were 90.58, 9.27 and 0.16% in the CAD group, compared with 88.72, 10.85 and 0.43% in the controls, respectively. The G allele frequency was 4.79% in the CAD group and 5.85% in the controls. There were no significant differences in genotype and allele frequencies between the CAD group and controls. Because the GG genotype was minor, AG and GG genotypes were grouped into the carriers of G allele (AG + GG). Crude OR with 95% CI and adjusted OR with 95% CI both indicated that carriers with G allele had not significant difference in the risk of CAD compared with carriers with AA genotype. Further subgroup analyses stratified by gender, age, EH and DM were carried out. No significant differences were found between the E670G polymorphism and CAD susceptibility in any subgroups (Table 3).

**Table 2:** Distribution in the genotype and allele frequencies of *PCSK9* E670G in CAD and control groups

Genotype/Allele	CAD [*n* (%)]	Controls [*n* (%)]	χ^2^	*P*
AA	567 (90.58)	417 (88.72)	1.474	0.478
AG	58 (9.27)	51 (10.85)		
GG	1 (0.16)	2 (0.43)		
A	1192 (95.21)	885 (94.15)	1.211	0.288
G	60 (4.79)	55 (5.85)		

*CAD* coronary artery disease

**Table 3:** Association of *PCSK9* E670G polymorphism and CAD risk

			CAD (n)	Controls (n)	Crude OR (95% CI)	*P*	Adjusted OR (95% CI)	*P* ^*@*^
All		AA	567	417	0.819 (0.553–1.212)	0.317	0.838 (0.548–1.280)	0.414
		AG + GG	59	53				
Gender	Male	AA	396	220	0.960 (0.564–1.636)	0.882	0.952 (0.541–1.675)	0.864
		AG + GG	43	23				
	Female	AA	171	197	1.636 (0.862–3.104)	0.132	0.706 (0.345–1.445)	0.341
		AG + GG	16	30				
Age	≥ 60 (years)	AA	407	268	0.850(0.530–1.365)	0.501	0.790(0.363–1.509)	0.407
		AG + GG	44	34				
	< 60 (years)	AA	159	149	0.740(0.363–1.509)	0.407	0.896(0.408–1.970)	0.785
		AG + GG	15	19				
EH	Yes	AA	415	244	0.717 (0.437–1.174)	0.186	0.688 (0.406–1.165)	0.164
		AG + GG	39	32				
	No	AA	152	173	1.084 (0.566–2.076)	0.808	1.260 (0.586–2.709)	0.554
		AG + GG	20	21				
DM	Yes	AA	149	57	0.946 (0.583–1.532)	0.820	0.497 (0.193–1.278)	0.147
		AG + GG	14	10				
	No	AA	418	360	0.901 (0.580–1.401)	0.644	0.536 (0.225–1.275)	0.158
		AG + GG	45	43				
Smoking	Yes	AA	219	105	1.065 (0.469–2.420)	0.880	1.055 (0.443–2.512)	0.903
		AG + GG	20	9				
	No	AA	348	312	0.795 (0.503–1.255)	0.325	0.834 (0.498–1.397)	0.490
		AG + GG	39	44				

*CAD* coronary artery disease, *OR* odds ratio, *CI* confidence interval

^@^Aadjustment with age, gender, smoking, EH, DM and lipid profiles

In addition, only GG genotype in R46L variant was detected in this population.

### Association between the *PCSK9* E670G polymorphism and lipid levels

The relationship between the *PCSK9* E670G polymorphism and lipid levels was also investigated. As shown in Table 4, serum lipid levels were not significantly different in carriers with G allele and those with AA genotype. Subgroup analyses based on age and gender did not find the association of the *PCSK9* E670G polymorphism with lipid profiles (Data were not listed).

**Table 4:** Association between *PCSK9* E670G polymorphism and lipid levels

		TC (mmol/l)	TG (mmol/l)	HDL-C (mmol/l)	LDL-C (mmol/l)	Apo A1 (g/l)	Apo B (g/l)	AIP
CAD	AA	4.59 ± 1.06	1.85 ± 1.53	1.08 ± 0.27	2.93 ± 0.93	1.18 ± 0.23	0.97 ± 0.30	0.15 ± 0.28
	AG + GG	4.64 ± 0.94	1.77 ± 1.15	1.07 ± 0.22	3.01 ± 0.78	1.19 ± 0.21	0.99 ± 0.26	0.05 ± 0.24
	*P*	0.565	0.876	0.798	0.462	0.574	0.457	0.339
Controls	AA	4.41 ± 0.97	1.77 ± 1.51	1.16 ± 0.30	2.64 ± 0.80	1.23 ± 0.24	0.91 ± 0.26	0.09 ± 0.34
	AG + GG	4.46 ± 0.90	1.63 ± 1.02	1.17 ± 0.34	2.67 ± 0.74	1.23 ± 0.24	0.90 ± 0.27	0.08 ± 0.36
	*P*	0.831	0.482	0.823	0.822	0.929	0.912	0.937

*CAD* coronary artery disease, *TC* total cholesterol, *TG* triglyceride, *HDL-C* high-density lipoprotein cholesterol, *LDL-C* low-density lipoprotein cholesterol, *AIP* atherogenic index of plasma

### Associations between serum PCSK9 levels and CAD risk and lipid levels

**Fig. 1:**
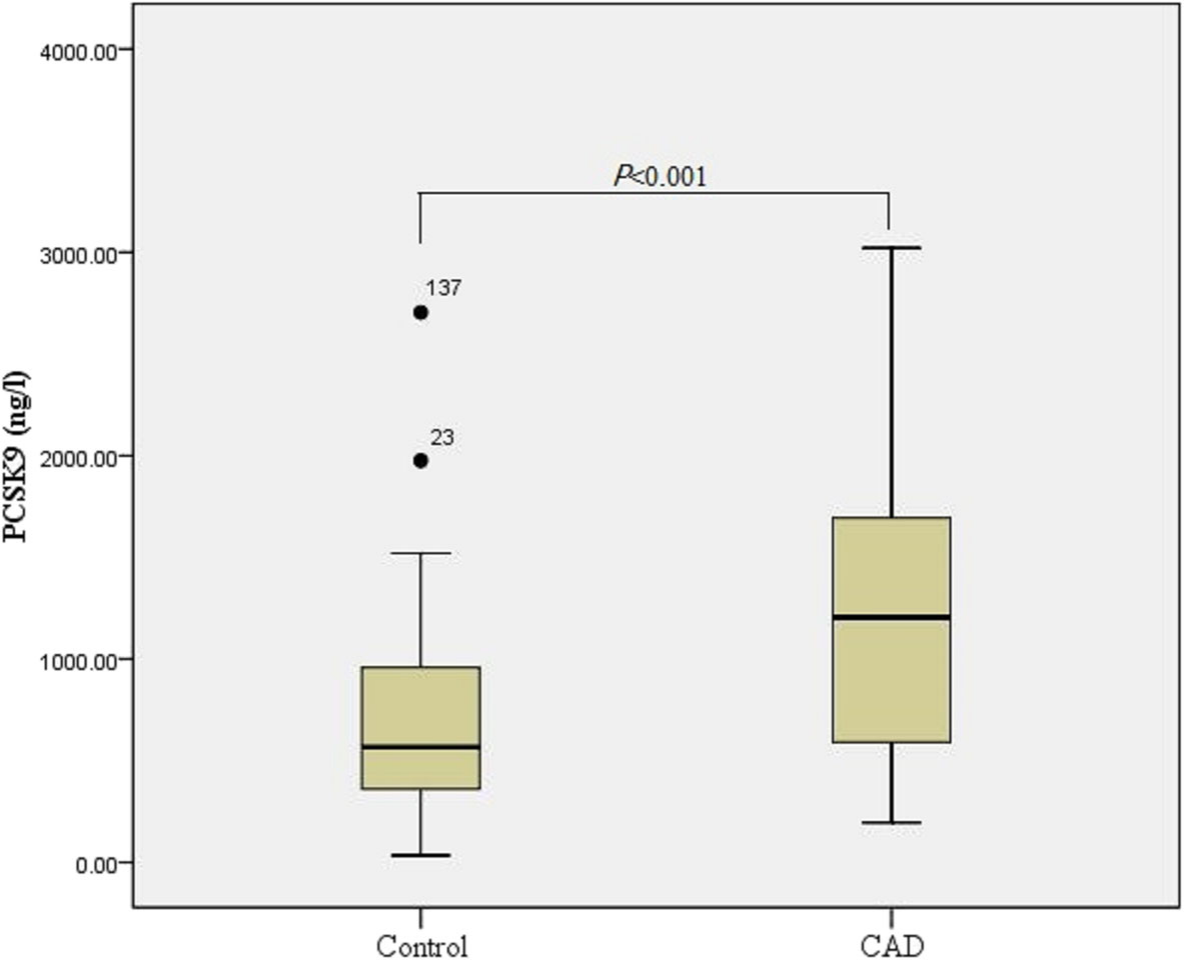
Serum PCSK9 levels in CAD and control groups

**Fig. 2:**
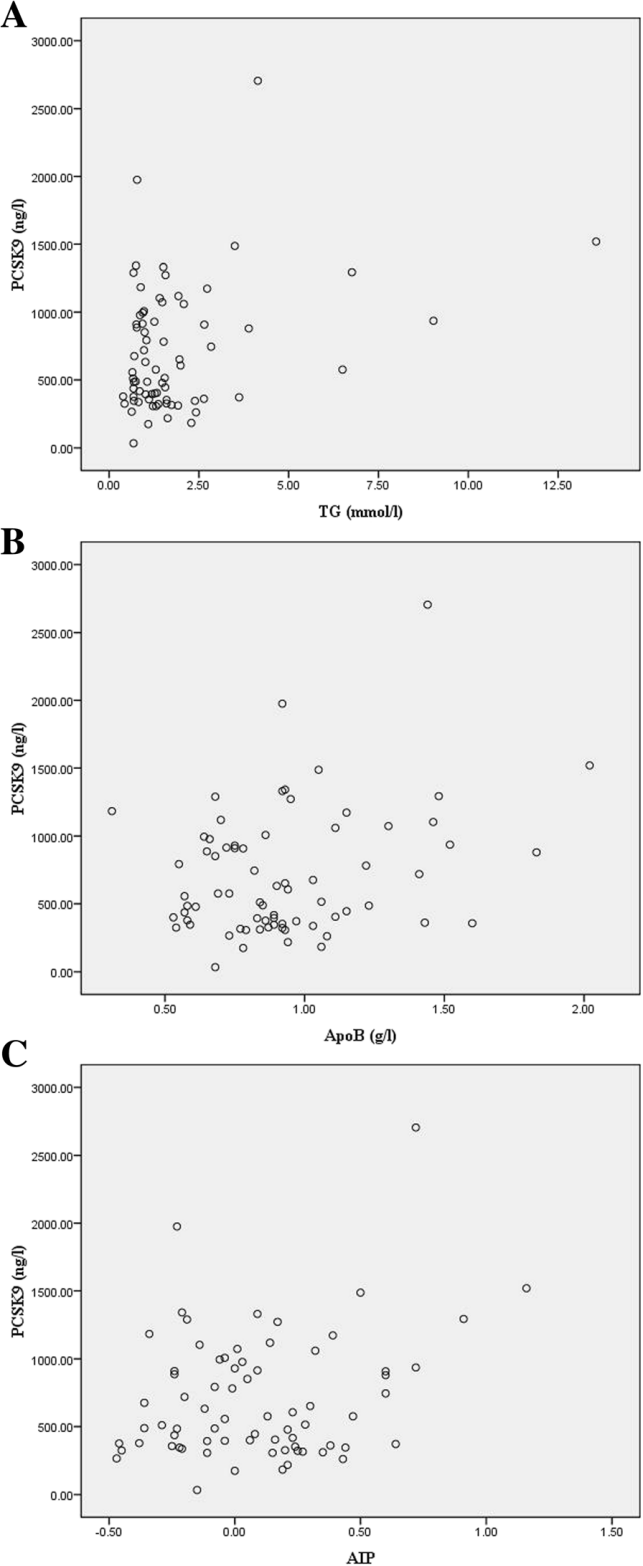
Correlation between PCSK9 levels and lipid profiles (**a** TG; **b** ApoB; **c** AIP)

144 subjects (72 cases and 72 controls) were selected to detect the serum PCSK9 level. There were no significant differences in age (CAD, age 65.26 ± 10.22 years; controls, age 62.47 ± 8.49 years, *P* > 0.05) and gender (CAD, male 68.06%; controls, male 58.33%, *P* > 0.05) between CAD and control groups. The median (QR) of PCSK9 concentration was 1205.00 ng/l (577.28–1694.13 ng/l) in cases and 565.87 ng/l (357.17–967.50 ng/l) in controls, respectively. Compared with controls, CAD patients had significantly higher PCSK9 levels (z = 4.559, *P* < 0.001) (Fig. 1). After adjusting with age, gender, EH, DM, smoking and lipid profiles, PCSK9 levels remain significantly associated with increased CAD susceptibility (OR = 1.002, 95% CI = 1.001–1.002, *P* < 0.001). The correlation analyses showed that serum PCSK9 levels were positively associated with TG, Apo B and AIP levels in the controls (Fig. 2, Table 5).

**Table 5:** Correlation analyses between PCSK9 levels and lipid profiles

		TC	TG	HDL-C	LDL-C	Apo A1	ApoB	AIP
Controls	r	0.13	0.32	−0.03	0.03	0.24	0.28	0.26
	*P*	0.28	*0.01*	0.80	0.83	0.05	*0.02*	*0.03*
CAD	r	0.07	−0.06	0.03	0.11	−0.01	0.02	−0.02
	*P*	0.59	0.63	0.82	0.37	0.95	0.85	0.87

*CAD* coronary artery disease, *TC* total cholesterol, *TG* triglyceride, *HDL-C* high-density lipoprotein cholesterol, *LDL-C* low-density lipoprotein cholesterol, *AIP* atherogenic index of plasma

### Association between *PCSK9* E670G polymorphism and PCSK9 levels

No significant association between *PCSK9* E670G polymorphism and PCSK9 levels was observed in CAD group and control group. Subgroup analyses based on age, gender, EH and DM status revealed that *PCSK9* E670G polymorphism was not associated with PCSK9 levels (Additional file 3: Figure S2).

## Discussion

PCSK9, originally named neural apoptosis regulated convertase-1 (NARC-1), is mainly expressed in the liver, kidney and intestine. In recent years, the associations between PCSK9 and the metabolism of LDL-C and the risk of CAD have been investigated extensively [5]. LDLR plays a crucial role in the metabolism of LDL-C in vitro and in vivo. In general, when LDL is combined with LDLR, it is internalized into clathrin-coated pits and subsequently degraded in the lysosome. LDLR is then recycled to the cell surface. PCSK9 increases the circulation unfriendly lipoprotein cholesterol by mediating the degradation and recirculation of several lipoprotein receptors, such as LDLR and VLDLR, via the EGF-A-binding domain. The inhibition of PSCK9 reduces LDL-C levels greatly, even in patients with statin ineffectiveness or intolerance [14], which has been seemed as a novel target for decreasing plasma lipid levels. Recent studies have indicated that PCSK9 increases the risk of CAD and is positively associated with CAD severity [15, 16]. In addition, circulating PCSK9 might independently predict the future risk of cardiovascular events [17]. The results of the present study indicated that PCSK9 levels were significantly higher in the CAD group than in the controls, which was consistent with previous reports describing the association between PCSK9 and the risk of CAD [5]. After adjusting for age, gender, EH, DM, smoking and lipid levels, PCSK9 levels remained an independent risk factor for CAD. The correlation analysis of PCSK9 and lipid profiles showed that PCSK9 levels were positively associated with TG and Apo B levels in the controls. Interestingly, PCSK9 levels were also positively associated with AIP, which was reported for the first time. Previous studies revealed that AIP, which represents the logarithm of the molar ratio of TG to HDL-C, was inversely associated with the diameter of LDL-C particles and indicated sdLDL particle size [18]. An increase of PCSK9 may predict a decrease in lipoprotein particle size, and this may partly explain the pro-atherosclerotic mechanisms of PCK9. After PCSK9 inhibition, the size of the LDL and VLDL particles increased, and the concentrations of LDL-C and total LDL particles were reduced, corroborating this speculation [19].

Previous studies have indicated that the *PCSK9* E670G polymorphism might be associated with lipid levels and the risk and severity of CAD [7]. This variant leads to a change from E to G at position 670 of the PCSK9 protein, and this may increase the affinity of PCSK9 for the LDLR. In the Strong Heart Family Study, a family-based genetic study, the authors found that E670G polymorphism was significantly associated with LDL-C levels in 2458 American Indians [20]. In Tunisian cohort, the 670G carriers had significantly higher TC and LDL-C levels compared to E670 carriers in CAD patients. In addition, the risk and severity of CAD were significantly increased in 670G carriers [21]. However, studies in different ethnic groups had controversial results [11, 22]. Even in the same ethnic population, the results were inconsistent, suggesting that region might affect the distribution of genotypes [23, 24]. Hsu LA et al. concluded that the *PCSK9* gene E670G polymorphism was not a risk factor for CAD, although it affects LDL-C levels [24]. However, Zhang N et al. revealed that carriers of the G allele had a higher risk of CAD than those with the A allele, and those with the G allele had higher TC and TG levels than those with the AA genotype in a Chinese population [25]. In the present study, only three subjects with the GG genotype were detected. The frequency of the minor allele was 5.25% in the whole population, consistent with the findings of Hsu LA et al. [24] and lower than those reported by Zhang N, et al. [25]. The findings of our study suggest that the *PCSK9* E670G polymorphism is neither associated with CAD susceptibility nor lipid profiles in the Southern Chinese Han population. Furthermore, subgroup analyses did not find any associations between the E670G polymorphism and CAD risk and lipid levels. The discrepancies of the associations between the *PCSK9* E670G polymorphism and the risk of CAD and lipid profiles among various populations may partly be due to the different characteristics of the studied populations, such as diagnostic criteria of CAD, ethnicity, region, and/or environmental factors. In 2013, Aung LH et al. found that the effects of the *PCSK9* E670G polymorphism on lipid profiles could be modified by alcohol consumption [26]. In the present study, we first investigated the relationship between the *PCSK9* E670G polymorphism and PCSK9 levels in the Chinese population, but no significant association was found. In addition, no *PCSK9* R46L polymorphism variant was detected in this Chinese population.

There were several limitations in this study. Firstly, age and gender between cases and controls were not matched, which might lead to the potential selection. Logistic analysis was used to reduce the bias as far as possible. Secondly, the present study was a hospital based case-control study, which may not be representative of the general population. Finally, the gene-environment interactions might affect the results. Environmental factors such as drinking status and degree of education therefore, might have influenced our results, which were not explored in our study.

## Conclusions

Despite its limitations, the present study shows that serum PCSK9 levels, but not *PCSK9* polymorphisms, are associated with the risk of CAD in Southern Chinese Han populations, and that serum PCSK9 levels are positively associated with AIP.

## Additional files

Additional file 1: **Table S1.** The sequence of PCR primers. **Table S2.** The sequence of probes used in the study. (DOCX 16 kb)

Additional file 2: **Figure S1.** The genemapper analyses of genotypes of *PCSK9* polymorphisms (A. E670G AA genotype; B. E670G AG genotype; C. E670G GG genotype; D. R46L GG genotype). (ZIP 7 kb)

Additional file 3: **Figure S2.** Associations between *PCSK9* E670G polymorphism and PCSK9 levels (A. in whole population; B. in case and controls subgroups; C. in male and female subgroups; D. in elderly and non-elderly subgroups; E. in EH and non-EH subgroups; F. in DM and non-DM subgroups). (ZIP 67 kb)

## Abbreviations

AIP: Atherogenic index of plasma
CAD: Coronary artery disease
CAG: Coronary angiography
DM: Diabetes mellitus
EH: Essential hypertension
HDL-C: High-density lipoprotein cholesterol
LDL-C: low-density lipoprotein cholesterol
PCSK9: Proprotein convertase subtilisin/kexin type 9
TC: Total cholesterol
TG: Triglyceride
