# Supplementary material for: Serum PCSK9 levels, but not PCSK9 polymorphisms, are associated with CAD risk and lipid profiles in southern Chinese Han population
Source: Lipids Health Dis. 2018 Sep 11;17:213. doi: 10.1186/s12944-018-0859-5 (PMC6134597; doi:10.1186/s12944-018-0859-5)
Supplement: Supplementary file 1 — Table S1. The sequence of PCR primers. Table S2. The sequence of probes used in the study. (DOCX 16 kb) [file 12944_2018_859_MOESM1_ESM.docx]

**Table S1 The sequence of PCR primers**

| Primer name | F sequence (5’-3’) | R sequence (5’-3’) | PCR length |
| --- | --- | --- | --- |
| rs505151 | CTACGCCGTAGACAACACGT | GCTGTCACTGGAGCTCCTG | 143 |
| rs11591147 | TGCTGCTGCTGCTCCTGGGT | CGGTGGAAGGTGGCTGTGGT | 141 |

**Table S2 The sequence of probes used in the study**

| Probe names | Sequence (5'-3’) | LDR length |
| --- | --- | --- |
| rs505151_modify | P-CTTCGCTGGTGCTGCCTGTAGTGCTTTTTTTTTTTTTTTTTTTTTTTTTTTTTTTTTTTTTTTTTTTTTTTTTTTTTTT-FAM |  |
| rs505151_A | TTTTTTTTTTTTTTTTTTTTTTTTTTTTTTTTTTTTTTTTTTTTTTTTTTGCAGATGGCAACGGCTGTCACGGCCT | 155 |
| rs505151_G | TTTTTTTTTTTTTTTTTTTTTTTTTTTTTTTTTTTTTTTTTTTTTTTTTTTTGCAGATGGCAACGGCTGTCACGGCCC | 157 |
| rs11591147_modify | P-GCAAGGCTAGCACCAGCTCCTTTTTTTTTTTTTTTTTTTTTTTTTTTTTTTTTTTTT-FAM |  |
| rs11591147_G | TTTTTTTTTTTTTTTTTTTTTTTTTTTTTTTTTTTTCGGCCAGGCCGTCCTCCTCGGAAC | 117 |
| rs11591147_A | TTTTTTTTTTTTTTTTTTTTTTTTTTTTTTTTTTTTTTCGGCCAGGCCGTCCTCCTCGGAAT | 119 |
| rs11591147_T | TTTTTTTTTTTTTTTTTTTTTTTTTTTTTTTTTTTTTTTTCGGCCAGGCCGTCCTCCTCGGAAA | 121 |
