# Supplementary figures and images for: Serum PCSK9 levels, but not PCSK9 polymorphisms, are associated with CAD risk and lipid profiles in southern Chinese Han population
Source: Lipids Health Dis. 2018 Sep 11;17:213. doi: 10.1186/s12944-018-0859-5 (PMC6134597; doi:10.1186/s12944-018-0859-5)

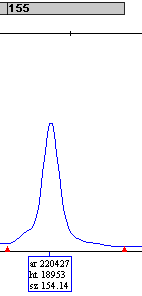

Supplement: Supplementary file 2 — Figure S1. The genemapper analyses of genotypes of PCSK9 polymorphisms (A. E670G AA genotype; B. E670G AG genotype; C. E670G GG genotype; D. R46L GG genotype). (ZIP 7 kb) [file 12944_2018_859_MOESM2_ESM.zip › S Fig 1A rs505151 AA.tif]

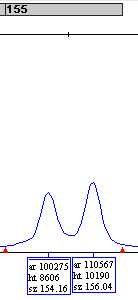

Supplement: Supplementary file 2 — Figure S1. The genemapper analyses of genotypes of PCSK9 polymorphisms (A. E670G AA genotype; B. E670G AG genotype; C. E670G GG genotype; D. R46L GG genotype). (ZIP 7 kb) [file 12944_2018_859_MOESM2_ESM.zip › S Fig 1B rs505151 AG.tif]

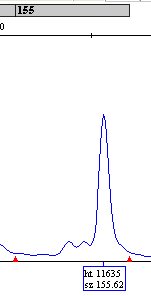

Supplement: Supplementary file 2 — Figure S1. The genemapper analyses of genotypes of PCSK9 polymorphisms (A. E670G AA genotype; B. E670G AG genotype; C. E670G GG genotype; D. R46L GG genotype). (ZIP 7 kb) [file 12944_2018_859_MOESM2_ESM.zip › S Fig 1C rs505151 GG.tif]

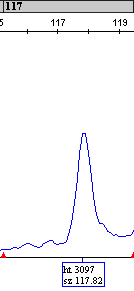

Supplement: Supplementary file 2 — Figure S1. The genemapper analyses of genotypes of PCSK9 polymorphisms (A. E670G AA genotype; B. E670G AG genotype; C. E670G GG genotype; D. R46L GG genotype). (ZIP 7 kb) [file 12944_2018_859_MOESM2_ESM.zip › S Fig 1D rs11591147 GG.tif]

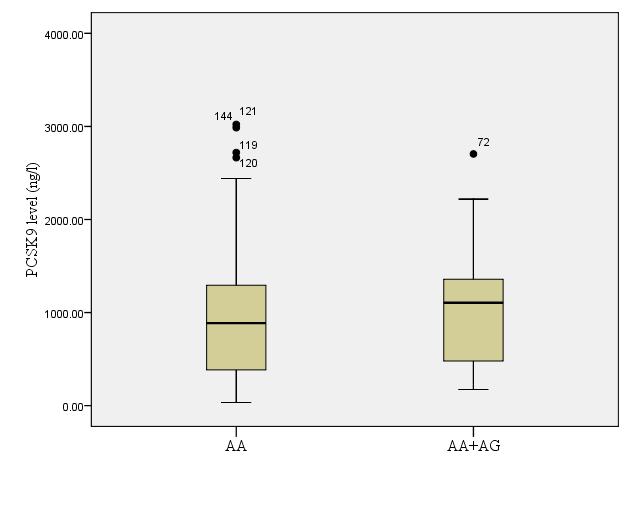

Supplement: Supplementary file 3 — Figure S2. Associations between PCSK9 E670G polymorphism and PCSK9 levels (A. in whole population; B. in case and controls subgroups; C. in male and female subgroups; D. in elderly and non-elderly subgroups; E. in EH and non-EH subgroups; F. in DM and non-DM subgroups). (ZIP 67 kb) [file 12944_2018_859_MOESM3_ESM.zip › S Fig 2A PCSK9 polymorphism PCSK9 levels in whole.jpg]

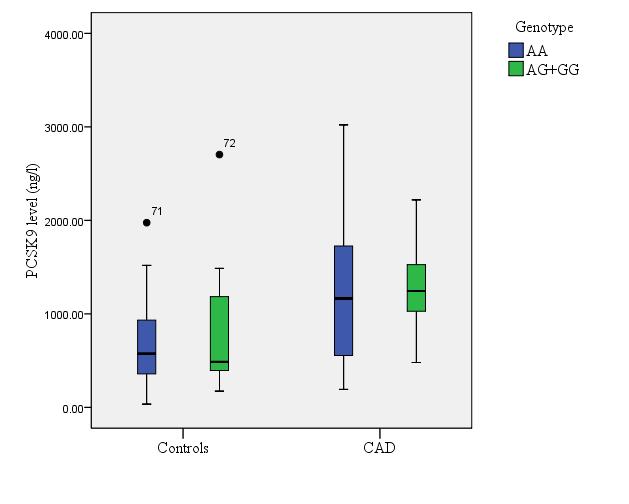

Supplement: Supplementary file 3 — Figure S2. Associations between PCSK9 E670G polymorphism and PCSK9 levels (A. in whole population; B. in case and controls subgroups; C. in male and female subgroups; D. in elderly and non-elderly subgroups; E. in EH and non-EH subgroups; F. in DM and non-DM subgroups). (ZIP 67 kb) [file 12944_2018_859_MOESM3_ESM.zip › S Fig 2B PCSK9 polymorphism PCSK9 levels in CAD.jpg]

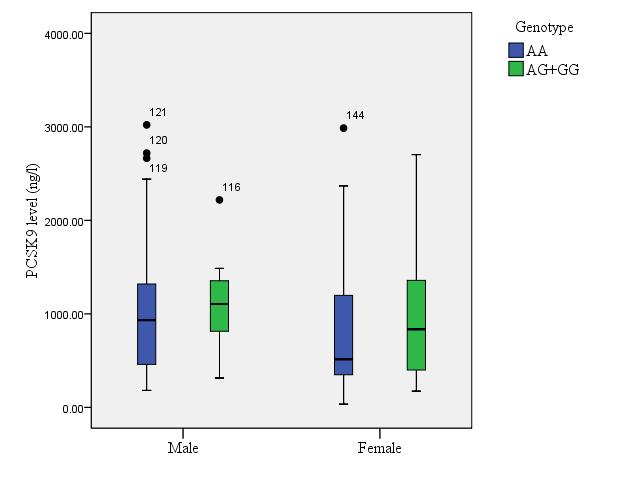

Supplement: Supplementary file 3 — Figure S2. Associations between PCSK9 E670G polymorphism and PCSK9 levels (A. in whole population; B. in case and controls subgroups; C. in male and female subgroups; D. in elderly and non-elderly subgroups; E. in EH and non-EH subgroups; F. in DM and non-DM subgroups). (ZIP 67 kb) [file 12944_2018_859_MOESM3_ESM.zip › S Fig 2C PCSK9 polymorphism PCSK9 levels in gender.jpg]

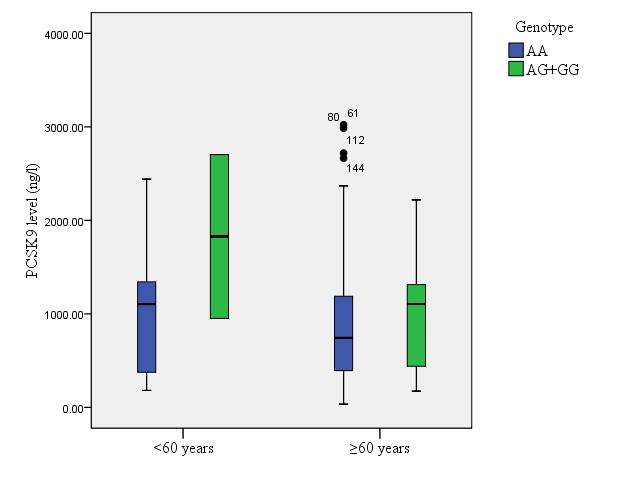

Supplement: Supplementary file 3 — Figure S2. Associations between PCSK9 E670G polymorphism and PCSK9 levels (A. in whole population; B. in case and controls subgroups; C. in male and female subgroups; D. in elderly and non-elderly subgroups; E. in EH and non-EH subgroups; F. in DM and non-DM subgroups). (ZIP 67 kb) [file 12944_2018_859_MOESM3_ESM.zip › S Fig 2D PCSK9 polymorphism PCSK9 levels in age.jpg]

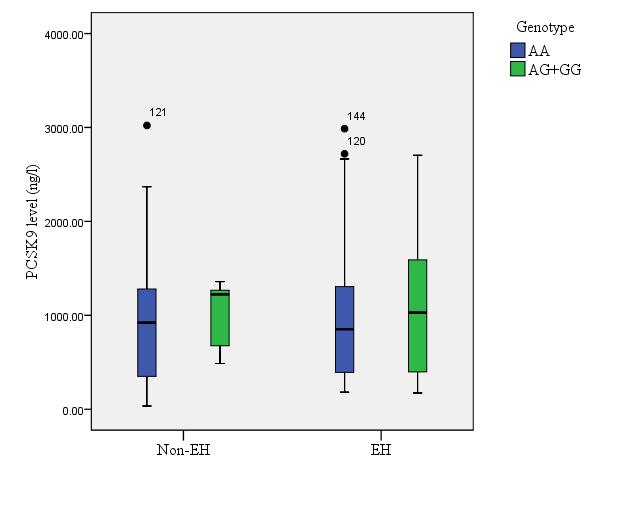

Supplement: Supplementary file 3 — Figure S2. Associations between PCSK9 E670G polymorphism and PCSK9 levels (A. in whole population; B. in case and controls subgroups; C. in male and female subgroups; D. in elderly and non-elderly subgroups; E. in EH and non-EH subgroups; F. in DM and non-DM subgroups). (ZIP 67 kb) [file 12944_2018_859_MOESM3_ESM.zip › S Fig 2E PCSK9 polymorphism PCSK9 levels in EH.jpg]

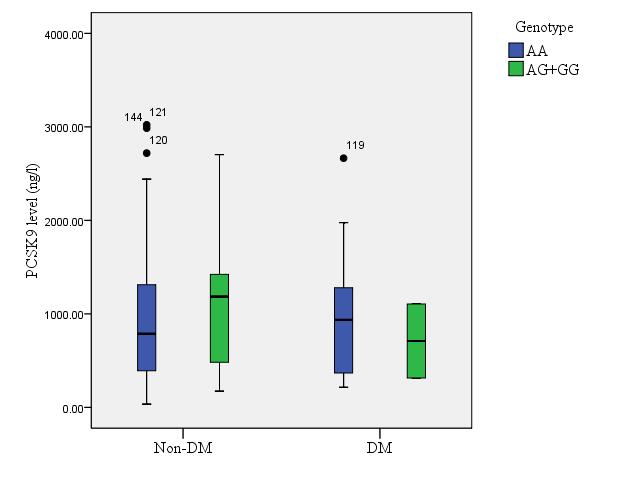

Supplement: Supplementary file 3 — Figure S2. Associations between PCSK9 E670G polymorphism and PCSK9 levels (A. in whole population; B. in case and controls subgroups; C. in male and female subgroups; D. in elderly and non-elderly subgroups; E. in EH and non-EH subgroups; F. in DM and non-DM subgroups). (ZIP 67 kb) [file 12944_2018_859_MOESM3_ESM.zip › S Fig 2F PCSK9 polymorphism PCSK9 levels in DM.jpg]
